# Urgent Anterior Cervical Osteophytectomy for an Asymptomatic Cervical Hyperostosis to Overcome Failed Intubation

**DOI:** 10.7759/cureus.2400

**Published:** 2018-03-31

**Authors:** Sultan Alsalmi, Abdulgadir Bugdadi, Abdu Alkhayri, Anthony Fichten, Johann Peltier

**Affiliations:** 1 Department of Neurosurgery, Amiens University Health Center, Amiens University, Amiens, FRA; 2 Department of Neurosurgery, Sorbonne University, Makkah Almukarramah, SAU; 3 Department of Neurosurgery, King Faisal Medical City, Abha, SAU

**Keywords:** cervical hyperostosis, forestier’s disease, dysphagia, lumbar canal stenosis, difficult intubation, diffuse idiopathic skeletal hyperostosis, dysphonia, odynophagia, change of voice

## Abstract

Cervical spondylosis and ankylosing hyperostosis of the cervical vertebrae are usually asymptomatic. This is a case report of a patient with massive anterior cervical osteophytes resulting in failure of intubation prior to a lumbar canal stenosis surgery. The osteophytes extended from C3 to C7 and resulted in the anterior displacement of the pharynx and the trachea. The patient was managed successfully with anterior cervical osteophytectomy.

## Introduction

Diffuse idiopathic skeletal hyperostosis (DISH), also known as Forestier’s disease, was first described by Forestier and Rotes-Querol in 1950 [1]. It is characterized radiologically by flowing calcification along the sides of the contiguous vertebrae of the spine. This ectopic calcification can lead to limitation of motion of the involved areas of the spine, which causes stiffness and dull pain. DISH is slowly progressive and the associated pain is usu­ally intermittent and thus overlooked and neglected by patients and physicians. Rarely, large bone spurs can form in front of the cervical vertebrae that can occasionally interfere with the passage of food. We present the case report of a male patient who underwent failed intubation as a result of such hyperostosis with chronic neck pain and dysphagia secondary to DISH, as well as to present a review of the literature.

## Case presentation

**Figure 1:**
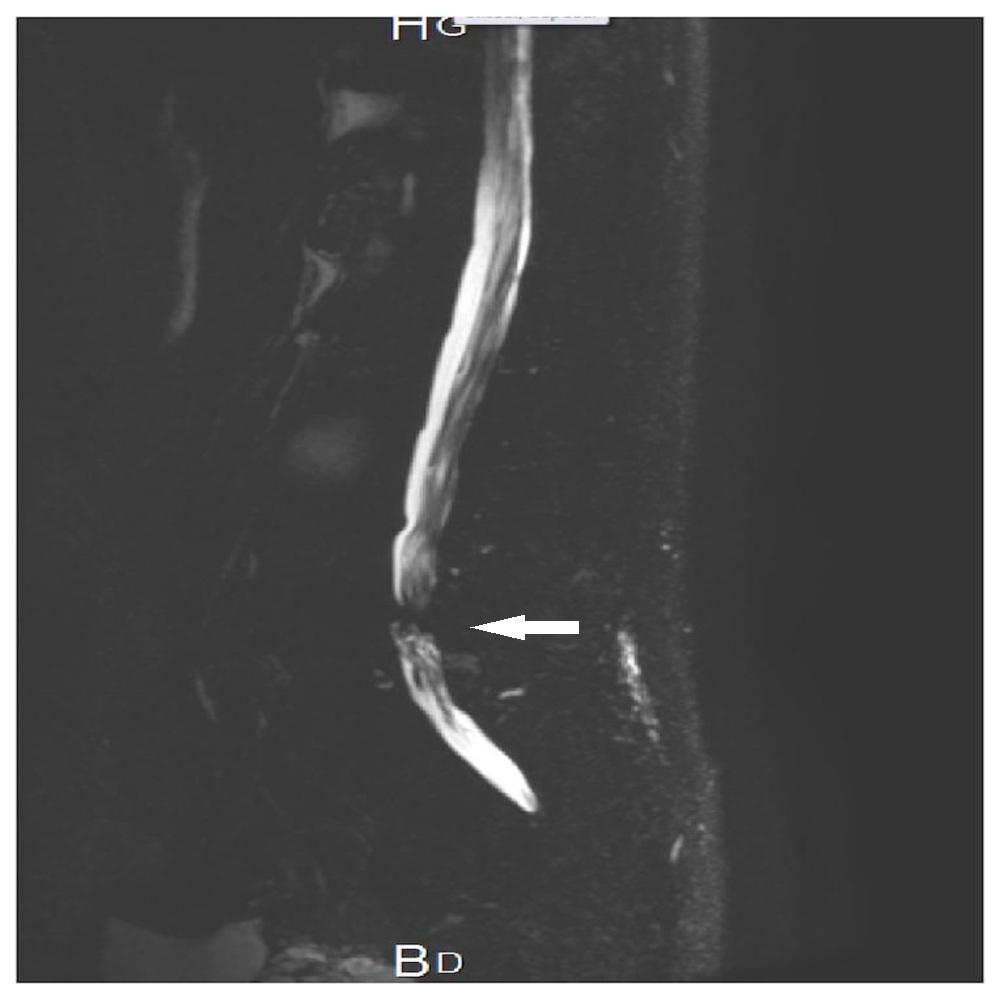
MR myelography of the lumbar spine showing severe L4-L5 lumbar canal stenosis (arrow) MR: magnetic resonance

A 66-year-old retired construction worker presented with chronic lower limb lumbosciatic pain bilaterally. The condition started 18 months prior to presentation with lumbago, followed by sciatic pain on the left side initially, and finally progressed to become bilateral. The patient had a history of Type 2 diabetes, hypercholesterolaemia, hemorrhagic rectocolitis, benign prostatic hypertrophy, and a right inguinal hernia. His past surgical history was positive for a surgically removed cervical lipoma in childhood. Physical examination was unremarkable. The patient had an average weight. Laboratory results weren’t significant, except for a microcytic hypochromic erythropoiesis. The complete blood count results were hemoglobin concentration (12.4 g/dl), hematocrit (35%), mean corpuscular volume (MCV) (78 fl), red blood cell (RBC) count (4,3*106µL), and mean corpuscular hemoglobin concentration (MCHC) (30 g/dL). Lumbar magnetic resonance imaging (MRI) showed severe lumbar canal stenosis, especially at the level of L4-L5 (Figure 1). The pre-anesthetic evaluation of the airway revealed easy visualization of the soft palate, fauces, uvula, and pillars (Mallampati class 2).

**Figure 2:**
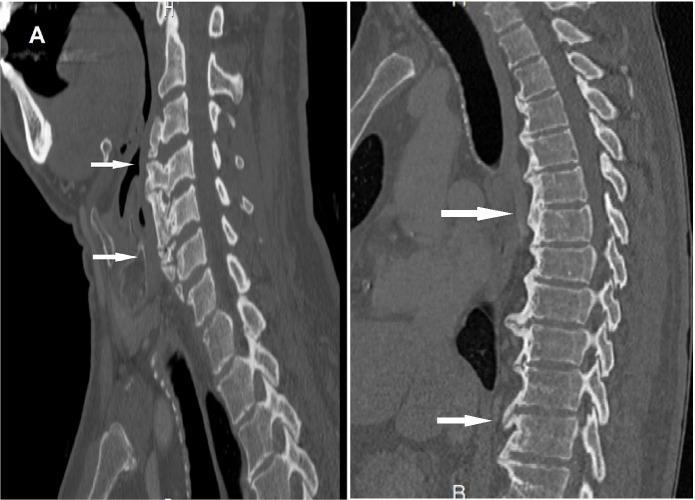
Preoperative sagittal cervical and thoracic computed tomography (CT) scan showing diffuse anterior hyperostosis. Arrows pointing to some large osteophytes. A) Sagittal cervical CT scan showing anterior hyperostosis and anteriorly deviated airway. B) Sagittal thoracic CT scan showing anterior hyperostosis.

The patient was booked for lumbar laminectomy to relieve the stenotic area, but the procedure was canceled due to failed trials of endotracheal intubation by two different anesthesiologists. When reporting this information to the patient, he declared having suffered from dysphagia, odynophagia, and hoarseness for a long time. A spinal CT scan was ordered which revealed extensive anterior vertebral bodies hyperostosis of the cervical and thoracic spine (Figure 2).

**Figure 3:**
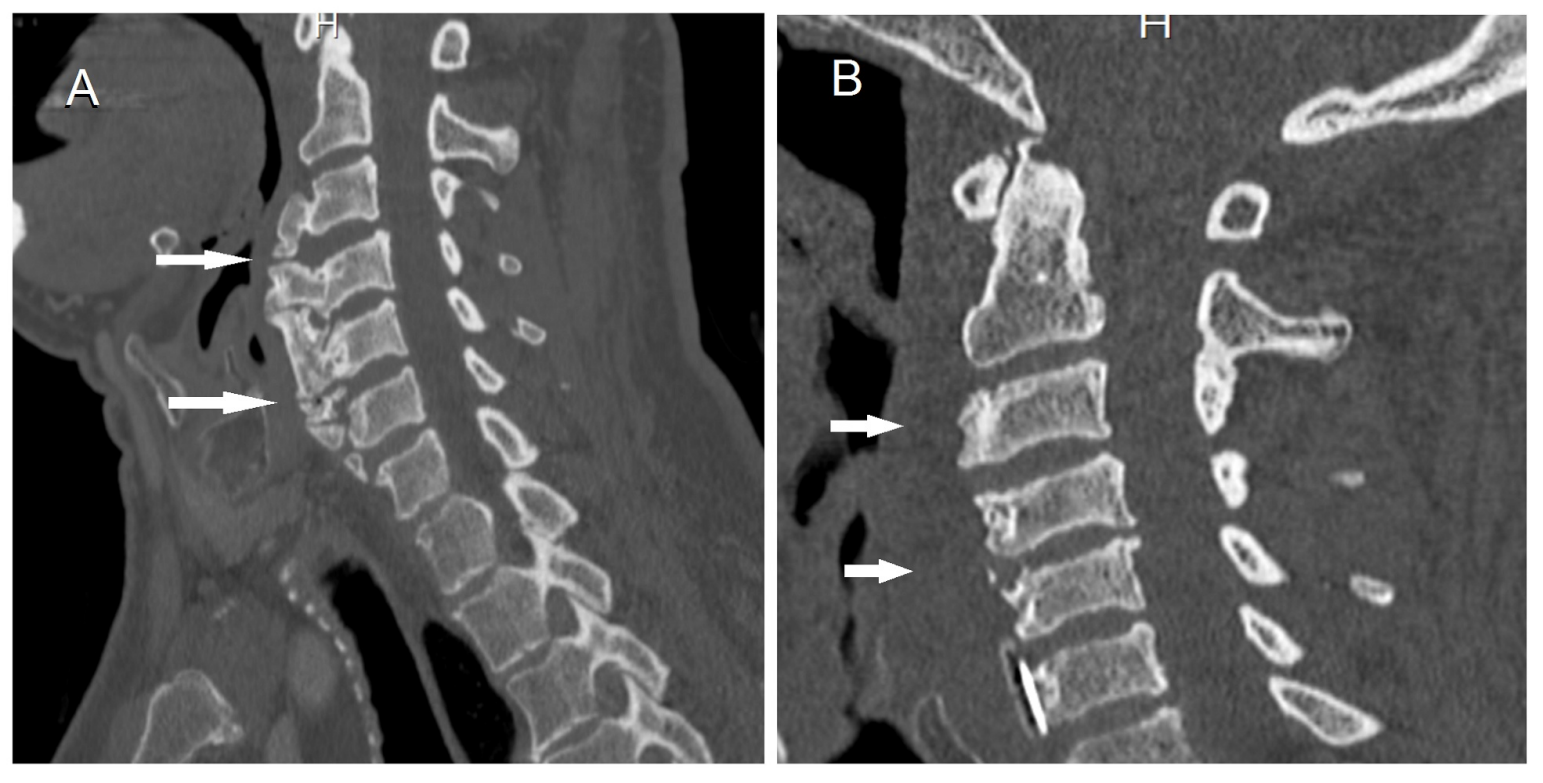
Sagittal cervical computed tomography (CT) scan. Arrows point to comparable regions. A) Preoperative sagittal cervical CT scan showing anterior hyperostosis. B) Post-osteophytectomy cervical CT scan

Following consultation with the anesthesiologist, the patient was planned for awake fiberoptic intubation and an informed consent was obtained. The patient was continually monitored through a sphygmomanometer, pulse oximeter, and electrocardiograph. Local anesthesia of the upper airway was accomplished with topical lidocaine spray followed by superior laryngeal nerve and transtracheal blocks. The patient was pre-oxygenated and 1 mg midazolam and 50 mcg fentanyl were slowly administered intravenously to achieve conscious sedation. His respiratory rate was 12 breaths per minute and oxygen saturation was greater than 95% on room air. The first attempt failed to achieve successful intubation, even with utilizing William’s airway, due to the expected presence of a large soft tissue mass extending from the posterior pharyngeal wall to the base of the tongue. On the second attempt, with patient’s cooperation, the epiglottis was noted to deviate to the left side while the vocal cords could only be visualized briefly with deep inspiration. Intubation was then successfully accomplished with 8.0 flexo-metallic cuffed endotracheal tube lubricated with lidocaine gel. An anterior cervical osteophytectomy extending from the level of C2-C3 till C7 was performed using a right semi-vertical paramedian cervical incision, guided by intraoperative fluoroscopy. Total anesthesia time was 45 minutes, while surgery time was 180 minutes (Figure 3).

## Discussion

Diffuse idiopathic skeletal hyperostosis (DISH), also known as Forestier's disease, is a disorder of unknown origin. It was first described by Forestier and Rotes-Querol in 1950 [1]. The disease is characterized by diffuse ossification and calcification of tendons, ligaments, and fasciae of both the axial and appendicular skeletons.

The disease has a predilection for men (65%) and is common in patients over the age of 50 years with a prevalence of approximately 15 - 20% in the elderly population [2-3]. The thoracic spine is most commonly involved (95% of cases),  followed by the lumbar and cervical spines [4].

According to Resnick [5], the radiographic diagnostic criteria in the spine include: 1) osseous bridging along the anterolateral aspect of at least four vertebral bodies; 2) relative sparing of intervertebral disc heights, with minimal or absent disc degeneration; and 3) absence of apophyseal joint ankylosis and sacroiliac sclerosis [5].

The classical surgical risks include hematoma, resection, or compression of the superior and/or inferior laryngeal nerves, salivary fistula, and esophageal perforation or infection [6]. The spine is usually characterized by ossification of the anterior longitudinal ligament resulting in fusion between at least four motion segments [7] with a variable degree of anterior ossification that could be very extensive. Secondary degenerative changes may occur in the posterior longitudinal ligament.

DISH is usually asymptomatic and lacks systemic inflammatory manifestations. If symptomatic, it can present in different ways. Some patients complain of neck pain or stiffness in the early phase [7]. Accelerated degeneration of the posterior annuli may result in prolapse, causing or adding to pre-existing central or foraminal symptoms [7]. Ossification of the posterior longitudinal ligament may also contribute to central canal stenosis. Extensive anterior ossification may result in compression of the pharynx and esophagus, causing dysphagia and/or dysphonia. In severe cases, patients may present with respiratory symptoms, such as recurrent respiratory tract infections or rarely, with acute respiratory distress. In case surgery is indicated, it is usually done electively to manage previously associated symptoms. To our knowledge, this is the first case described where DISH was associated with intubation difficulties, necessitating surgery in order to facilitate further management of other conditions.

Removal of the anterior osteophytes typically requires a more extensive exposure than that for anterior cervical discectomy and fusion or cervical corpectomy. The long incision, to access the lower cervical segments, enables access to all the bony spurs but risks damage to the recurrent laryngeal nerve that may already be under considerable tension. Commonly, a right-sided approach is preferred to lessen the risk of iatrogenic recurrent laryngeal nerve injury [8].

The surgeon should carefully remove the abnormal tissues and not breach the intact anterior annulus, risking iatrogenic destabilization. Typically, there is a cleavage plane between the vertebral body and pathological tissue [9]. Intraoperative fluoroscopy is helpful in determining the degree of bony resection. Standard spinal haemostatic agents, and particularly bone wax, are useful in the control of bleeding. The use of a postoperative drain is recommended because of the significant risk of developing a postoperative hematoma. The surgical outcome is generally very good. Neurological decompression will follow the expected postoperative course with radiculopathy recovering more predictably than established myelopathy.

Dysphagia recovers very early. One case series, including 12 patients with six to 60 months of follow-up, concluded that dysphagia recovers within one month postoperatively. One patient required revision of the anterior decompressive surgery for recurrent osteophyte formation [10].

In our case, due to the fact that the patient’s airway was already narrowed from the osteophyte formation, we chose to carry out a cervical osteophytectomy prior to pursuing the lumbar laminectomy. We felt that intubation in an already compromised and narrowed airway was an unnecessary risk for complications, such as airway edema following intubation for the lumbar laminectomy, without first addressing the osteophyte formation in the cervical spine. In our point of view, we believe that this is a reasonable approach to deal with such a situation. Following this strategy, we managed the patient’s complaints and eliminated a possible fatal difficulty.

## Conclusions

Cervical osteophytes are likely to be missed in a routine airway examination. A high index of suspicion is required to anticipate this condition, mainly if there is a history of dysphagia, dyspnea, sensation of a lump in the throat, or a change in the character of voice. In such cases, a cervical CT scan should be considered. Surgical excision of the osteophytes via anterior approach is an effective method of treatment for such patients before dealing with the other non-emergent surgical conditions to secure the airway and facilitate emergent intubation in case it is needed.
